# Interactive effects of early and recent exposure to stressful contexts on cortisol reactivity in middle childhood

**DOI:** 10.1111/jcpp.12287

**Published:** 2014-07-01

**Authors:** Sara R Jaffee, Tara McFarquhar, Suzanne Stevens, Isabelle Ouellet-Morin, Edward Melhuish, Jay Belsky

**Affiliations:** 1 Department of Psychology, University of PennsylvaniaPhiladelphia, PA, USA; 2 Social, Genetic, and Developmental Psychiatry Centre, King's College LondonLondon, UK; 3 Psychology and Language Sciences, University College LondonLondon, UK; 4 Anna Freud CentreLondon, UK; 5 Department of Psychological Medicine, University of AucklandAuckland, New Zealand; 6 School of Criminology, Université de MontréalMontreal, QC, Canada; 7 Mental Health Institute of Montréal Research CenterMontreal, QC, Canada; 8 Research Group on Child MaladjustmentMontreal, QC, Canada; 9 The Department of Psychological Sciences, Birkbeck, University of LondonLondon, UK; 10 Department of Education, University of OxfordOxford, UK; 11 Department of Psychology, University of WollongongWollongong, NSW, Australia; 12 Department of Human Ecology, University of CaliforniaDavis, CA, USA

**Keywords:** Cortisol reactivity, stress, parenting, internalizing, externalizing

## Abstract

**Background:**

Given mixed findings as to whether stressful experiences and relationships are associated with increases or decreases in children's cortisol reactivity, we tested whether a child's developmental history of risk exposure explained variation in cortisol reactivity to an experimentally induced task. We also tested whether the relationship between cortisol reactivity and children's internalizing and externalizing problems varied as a function of their developmental history of stressful experiences and relationships.

**Method:**

Participants included 400 children (*M* = 9.99 years, *SD* = 0.74 years) from the Children's Experiences and Development Study. Early risk exposure was measured by children's experiences of harsh, nonresponsive parenting at 3 years. Recent risk exposure was measured by children's exposure to traumatic events in the past year. Children's cortisol reactivity was measured in response to a social provocation task and parents and teachers described children's internalizing and externalizing problems.

**Results:**

The effect of recent exposure to traumatic events was partially dependent upon a child's early experiences of harsh, nonresponsive parenting: the more traumatic events children had recently experienced, the greater their cortisol reactivity if they had experienced lower (but not higher) levels of harsh, nonresponsive parenting at age 3. The lowest levels of cortisol reactivity were observed among children who had experienced the most traumatic events in the past year and higher (vs. lower) levels of harsh, nonresponsive parenting in early childhood. Among youth who experienced harsh, nonresponsive parent–child relationships in early childhood and later traumatic events, lower levels of cortisol reactivity were associated with higher levels of internalizing and externalizing problems.

**Conclusions:**

Hypothalamic–pituitary–adrenal (HPA) axis reactivity to psychological stressors and the relationship between HPA axis reactivity and children's internalizing and externalizing problems vary as a function of a child's developmental history of exposure to stressful relationships and experiences.

## Introduction

Biopsychosocial models propose that early exposure to harsh, dangerous, or unpredictable environments increases risk for poor mental and physical health by adversely affecting the development of central nervous, neuroendocrine, and immune systems (Danese & McEwen, 2012). Consistent with this possibility, children from families characterized by harsh, rejecting, and conflictual relationships and children who are exposed to violence show different patterns of hypothalamic–pituitary–adrenal (HPA) axis functioning than children who do not experience these stressful relationships or experiences (Tarullo & Gunnar, 2006). As we describe below, adverse developmental experiences and environmental exposures have been linked to both hyper- and hypo-reactivity. Relatively little is known, however, about whether the timing and chronicity of exposure to stress affect children's HPA axis functioning (Hostinar & Gunnar, 2013). The primary goal of the current study was to test whether individual differences in children's HPA axis reactivity vary as a function of their developmental history of exposure to stressful relationships and experiences.

Studies of HPA axis reactivity have typically focused on exposure to stressful relationships or experiences at specific points in time, without considering the possibility that the effects of a stressor at one point in time may be dependent on exposure to a stressor at another point in time. For example, two studies focused on the effects of maltreatment in early (Gunnar, Frenn, Wewerka, & Van Ryzin, 2009) or middle childhood (Shenk, Noll, Putnam, & Trickett, 2010) on HPA axis reactivity to psychosocial stress approximately 6–10 years later. Both of these investigations revealed that maltreated youth had *lower* levels of cortisol reactivity than comparison youth, although neither study accounted for the possible continuity of exposure to stressful relationships or experiences on HPA axis function.

Two other inquiries focused solely on more recent exposure to psychosocial stressors, without accounting for the possible effects of early exposure. For example, MacMillan et al. (2009) found that 12- to 16-year-old girls who had open cases with Child Protective Services showed *lower* levels of cortisol reactivity to a psychosocial stressor than comparison girls. Similarly, in a sample of 8- to 13-year-olds, those who had been exposed to violence in the past year had significantly *lower* levels of cortisol reactivity than those who were not violence-exposed (Marsman et al., 2012).

The timing of exposure to psychosocial stressors is difficult to determine in still other research. For example, in two studies in which exposure to bullying (Ouellet-Morin, Danese, et al., 2011) or family aggression (Saxbe, Margolin, Shapiro, & Baucom, 2012) may have occurred at any point (or consistently) over several years, those who were bullied and those who experienced higher levels of family aggression had *lower* levels of cortisol reactivity to psychosocial stressors in early to midadolescence than those who were nonbullied or who experienced lower levels of family aggression. In contrast, two other investigations found that adolescents who reported a lifetime history of maltreatment had significantly *higher* cortisol reactivity to a psychosocial stressor than adolescents who did not have a lifetime history of maltreatment (Harkness, Stewart, & Wynne-Edwards, 2011; Linares, Shrout, Nucci-Sack, & Diaz, 2013). To our knowledge, the only research that specifically evaluated whether there were interactive effects on adolescents' cortisol reactivity of familial adversity at different points across childhood and adolescence failed to detect significant effects (Bosch et al., 2012).

Although the cited work generally reveals a pattern of blunted cortisol reactivity among youth who were victimized or exposed to familial aggression, individuals who vary in their developmental history of exposure to stressful relationships or experiences may have markedly different patterns of physiological reactivity to lab-induced stressors (Del Giudice, Ellis, & Shirtcliff, 2011). We hypothesized that with consistent exposure to harsh, rejecting, or dangerous relationships and environments across early and middle childhood, the cortisol response to experimentally induced stressors would be relatively low. In contrast, we hypothesized that in the absence of an early childhood history of harsh, nonresponsive parenting, recent exposure to traumatic events would be associated with increased cortisol reactivity.

A second goal of the research was to test whether the relationship between cortisol reactivity and children's internalizing and externalizing problems varied depending on children's history of stressful relationships and experiences – a necessary step in identifying the clinical implications of the biological embedding of early experiences. Consistent with a diathesis–stress model, HPA axis reactivity is more strongly associated with child and adolescent problem behaviors when youth have had stressful relationships and experiences than when youth have been free of such stressors. The direction of this association varies across studies, however, with some work indicating that low levels of cortisol reactivity *increase* risk for emotional and behavioral problems (Badanes, Watamura, & Hankin, 2011; von Klitzing et al., 2012; Ouellet-Morin, Odgers et al., 2011) and other research finding that low levels of cortisol reactivity have *buffering* effects on children's mental health (von Klitzing et al., 2012; Saxbe et al., 2012).

Given the centrality of the early parent–child relationship as a context for children's social and emotional development (Gunnar & Quevedo, 2007; Sroufe, 2005), we defined early risk exposure as higher (vs. lower) levels of harsh and nonresponsive parent–child relations at age 3 years. Because children are exposed to a broader set of influences over time, the measure of recent risk exposure comprised traumatic events that children experienced both inside and outside the family in the year prior to the data collection. We hypothesized that the effect of recent exposure to traumatic events would depend on a child's early experience of parenting and that cortisol reactivity would be more strongly associated with children's internalizing and externalizing problems if children had been exposed to stressful relationships and experiences over time.

## Methods and materials

### Sample

The sample included 400 children (51% male) who participated in the Children's Experiences and Development Study (CEDS), which was conducted from 2009 through 2011 in England. CEDS children were born between 1999 and 2001 and were originally assessed as part of a separate study of over 6,000 families when they were 3 years old (Belsky, Melhuish, Barnes, Leyland, & Romaniuk, 2006). Participants ranged from 8 to 11 years (*M* = 9.99, *SD* = 0.74) and were predominantly White (70%), with the remainder South Asian (16%), African or Afro-Caribbean (7%), or of other ethnicities (7%). Mean annual pretax household income corresponded to £18,000–£19,000 (approximately USD $29,000–$30,500). On average, the highest educational attainment in the household was an O-level qualification or equivalent, corresponding to secondary school completion.

Families were recruited to CEDS on the basis of children's experiences of harsh and nonresponsive parenting when they were 3 years old (measures described below). CEDS families were selected on this basis because the combination of high levels of harsh and low levels of responsive parenting is often associated with the most adverse behavioral and cognitive outcomes for children (Caron, Weiss, Harris, & Catron, 2006; Deater-Deckard, Ivy, & Petrill, 2006; German, Gonzales, McClain, Dumka, & Millsap, 2013; McKee et al., 2007; McLoyd & Smith, 2002; Smith & Brooks-Gunn, 1997). Thus, we sampled a ‘higher risk’ group that included children who had experienced relatively high levels of harsh, nonresponsive parenting at age 3 years (in the top tertile of the harsh discipline composite and below the median on the responsive parenting measure; cut-points for measures varied as a function of the measures' distributions). A ‘lower risk’ group included children who had experienced relatively low levels of harsh, nonresponsive parenting at age 3 years (in the bottom two tertiles of the harsh discipline composite and above the median on the responsive parenting measure). The sampling frame is depicted in Figure S1 (available online). Differences (when the children were 3 years old) between the 400 families who participated in CEDS and the 729 families who could not be located or who refused to participate are presented in Table S1 (available online). Housing and employment stability may have made it easier to locate the families who ultimately participated in CEDS.

### CEDS protocol

Week-long training sessions were held before research workers went into the field. Visits were conducted in the family's home and lasted approximately 4 hr. Visits were usually scheduled for weekday afternoons and involved an interview with the child and with the child's main caregiver (i.e., mother in 98% of families). Research workers obtained signed consent from caregivers and signed and/or verbal assent from children before beginning the interview. Store vouchers were paid to caregivers (£35) and children (£10) for their participation. Ninety-seven percent of parents provided contact details for their child's teacher and 70% of teachers who were contacted completed questionnaires about the child's behavior. Teachers were given a £10 store voucher for their participation. The study was approved by the King's College London Research Ethics Committee.

### Measures

*Harsh parenting at age 3 years*. Using the Home Observation for Measurement of the Environment (HOME; Caldwell & Bradley, 1984), research workers indicated whether caregivers engaged in any of three physically or verbally harsh behaviors during the home visit. Responses to these items were summed (*M* = 0.21, *SD* = 0.54). Caregivers responded to eight items from the Conflict Tactics Scale: Parent–Child (CTS-PC; Straus, Hamby, Finkelhor, Moore, & Runyan, 1998) about how often in the past year (0 ‘never’ to 6 ‘more than 20 times’) they used verbally or physically harsh disciplinary techniques. The CTS-PC items were summed (*M* = 14.86, *SD* = 9.90, α =.77). The CTS-PC and HOME measures of harsh discipline were standardized and averaged to create a harsh discipline composite score that was approximately normally distributed (*M* = 0.00, *SD* = 0.75).

*Responsive parenting at age 3 years* was assessed using eight questions from the Responsive Parenting subscale from the HOME about behavior that research workers witnessed in the home (e.g., ‘mother caresses, kisses, or cuddles child at least once during visit’). Each item was coded as either observed or not observed and responses were summed (*M* = 4.98, *SD* = 1.53).

*Recent traumatic events* (at follow-up) were measured with the Traumatic Events Screening Inventory (Ribbe, 1996). Youth and caregivers were asked whether any of 13 traumatic events had ever happened to the child and, if so, had happened in the past year. Because agreement between caregiver and child reports was modest (kappas ranged from 0 for low base rate events like ‘child was kidnapped’ to.51 for ‘family member was in trouble with the police or in prison’), events were coded as having happened only if both the child and caregiver reported the event (*M* = 0.61, *SD* = 0.85). In the past year, 58% of children experienced no traumatic events, 27% one event, and 15% 2–4 events. The most common (past-year) traumatic events reported were (a) hearing ‘people outside your home really yelling and screaming at each other a lot’ (24%), (b) hearing ‘people inside your family really yelling and screaming at each other a lot’ (11%), and (c) witnessing violence outside the home or in the neighborhood (e.g., people beating each other up, fighting or attacking, shooting, stabbing; 9%).

*Internalizing and externalizing problems (at follow-up)* were reported by caregivers and teachers using a modified version of the Child and Adolescent Symptom Inventory-4R (Gadow & Sprafkin, 2005) that included a subset of scales. Internalizing problems comprised symptoms of generalized anxiety disorder (GAD) and dysthymia. Externalizing problems comprised symptoms of oppositional defiant disorder (ODD) and conduct disorder (CD). A symptom was considered present if the child engaged in the behavior ‘often’ or ‘very often’ in the case of more common behaviors or if the child ever engaged in less common behaviors. Symptoms of each disorder were summed. Parent (and teacher) reports of internalizing problems were formed by standardizing and averaging the GAD and dysthymia scores and parent (and teacher) reports of externalizing problems were formed by standardizing and averaging the CD and ODD scores. The Pearson correlations between caregiver and teacher reports were *r* =.37, *p* <.001 for internalizing problems and *r* =.42, *p* <.001 for externalizing problems. Caregiver and teacher scores were standardized and averaged together to create cross-informant internalizing (*M* = −0.01, *SD* = 0.86, α =.80) and externalizing scores (*M* = −0.01, *SD* = 0.88, α =.91).

*Social provocation task (at follow-up)*. The social provocation (SP) task was designed to elicit feelings of frustration and anger in children and was modified from a task developed by van Goozen et al. (1998). The SP task was introduced approximately 55 min into the visit with the child and lasted for approximately 30 min. Children were told they were going to play a video game for a £10 prize against an online opponent who would be visible via a webcam and who would observe the participant's performance. The opponent was actually a confederate who had been previously video-recorded participating in the SP task. The opponent was the same age as the participant and matched for sex.

The game included a practice round (2 min), two competitive rounds (each 3 min), and a final round played by the opponent (3 min). The game was rigged so that participants performed poorly in each round and were outscored by the opponent in the final round, despite opportunities to hinder the opponent's performance. Each round was followed by a (prerecorded) video message from the opponent that included derogatory and taunting comments about the participant's performance in the game.

Upon conclusion of the SP task, participants spent approximately 5 min completing an easy task designed to afford success. At the conclusion of the study, children and parents were debriefed by research workers who informed them that the game had been rigged. No child indicated that she or he had been aware of the deception.

*Cortisol* was assayed from saliva samples that were obtained via passive drool. Salimetrics™ cryovials (polypropylene, 2 ml capacity) were used to collect at least 1 ml of passive drool from participants. Children were instructed to refrain from vigorous exercise or from eating snacks or dairy products after midday on the day of testing. Saliva was collected at 20 and 10 min prior to the start of the SP task, at the end of the SP task, and at approximately 20, 45, and 65 min following the conclusion of the SP task. Nineteen percent of children refused to participate in round 2 of the SP task (often because they were very distressed). For these children, the timing of the saliva collection was identical to that described above, except they lacked the sample collected at the end of the SP task. Samples were refrigerated and then frozen at −20°C.

Samples were assayed using the Salimetrics™ Cortisol hormone kit, which is a competitive immunoassay specifically designed and validated for the quantitative measurement of salivary cortisol. Samples were thawed for 15 min and then centrifuged at 3000 rcf. Optical density was read on a microplate spectrophotometer at 450 nm with a correction of 620 nm. The samples were measured in duplicate, with none of the optical density coefficients of variation exceeding 10%. The assay had an analytical sensitivity of 0.003 μg/dl. Cortisol values were inspected for outliers (falling 3 or more standard deviations away from the mean); outliers were winsorized to the highest nonoutlier value. Natural log transformations were conducted to normalize the winsorized cortisol concentration distributions.

*Self-reported emotion (at follow-up)*. To check that the SP task was frustrating or distressing, children were asked 10 questions about how they were feeling immediately before the practice round, after the practice round, after round 1, and after the opponent's round. Children responded on a scale from 1 ‘not at all’ to 5 ‘extremely.’

*Control variables*. The time that children went to bed the previous evening (*M* = 9:32 pm, *SD* = 1.21), medication status (17% of children regularly took some medication including steroidal inhalers), and the time saliva sampling started (*M* = 15:05, *SD* = 1.62 hr) were included as control variables because these were correlated with some cortisol values. Analyses controlled for child (sex, age, race) or family characteristics (income, education, and occupational status) if they were correlated with the outcome variable or with the measure of cortisol reactivity (Table S2 for bivariate correlations). Although caregivers reported on children's pubertal status using the Pubertal Development Scale (Petersen, Crockett, Richards, & Boxer, 1988), we did not include pubertal status as a covariate because it was not significantly correlated with either the individual cortisol values or cortisol reactivity (for individual cortisol values, maximum *r* =.03, *p* =.72; for cortisol reactivity, *r* = −.01, *p* =.84).

## Results

### Was the social provocation task stressful for children?

Because cortisol concentrations at time *t* reflect cortisol activity *t –* 15 to *t –* 30 min earlier, *cortisol reactivity* to the SP task was assessed by subtracting the lowest cortisol value collected by the end of the SP task (which reflected cortisol activity prior to or in the very early stages of the SP task) from the cortisol value collected 20 min following the SP task (which reflected cortisol activity at the conclusion of the SP task). This approach was preferable to averaging the cortisol values that were gathered before the end of the SP task because the values tended to fall in a linear fashion from a relatively high level, perhaps reflecting the initial novelty of the research worker's visit. Moreover, given that the final cortisol value collected by the end of the SP task may have reflected cortisol concentrations shortly before the beginning of the SP task for some individuals and may have reflected cortisol concentrations in the early stages of the SP task for others, it was not considered a good universal baseline measure. The minimum pretask and 20-min posttask cortisol values are presented in Table S3. Pairwise *t*-tests showed that regardless of whether youth completed round 2, cortisol values increased significantly from the minimum value prior to the SP task to the value at 20 min following the SP task when cortisol levels were expected to peak (round 2 completed: *t* [314] = −5.21, *p* <.001; round 2 not completed: *t* [65] = −5.37, *p* <.001). Thus, these groups were combined.

Mixed effects ANOVA (controlling for between-subjects effects of child sex, age, and race) showed that children became increasingly frustrated, *F* (3, 912) = 39.77, *p* <.001, angry, *F* (3, 912) = 61.26, *p* <.001, embarrassed, *F* (3, 912) = 15.87, *p* <.001, and felt decreasingly in control, *F* (3, 900) = 20.37, *p* <.001 as the task proceeded. Change in self-reports of emotions did not vary as a function of child sex, age, or ethnicity.

### Does a developmental history of exposure to stressful relationships and experiences account for variation in cortisol reactivity?

**Figure 1:**
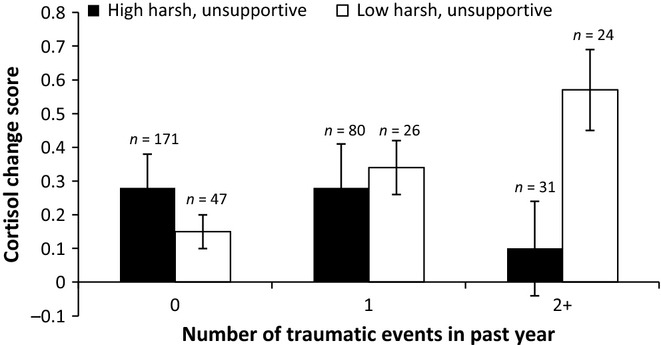
Harsh, nonresponsive parenting and recent traumatic events interact to predict cortisol reactivity (error bars are standard errors)

An ordinary least squares regression analysis was conducted in which cortisol reactivity values were regressed on harsh, nonresponsive parenting, traumatic events, and covariates (medication status, the previous night's bedtime, and time of initial saliva sampling) at the first step and on the interaction between harsh, nonresponsive parenting and traumatic events at the second step. All variables were mean-centered prior to analysis. The main effect of recent traumatic events on cortisol reactivity was qualified by a significant interaction involving harsh, nonresponsive parenting (Table 1 and Figure 1). Results were unchanged, controlling for participation in round 2 of the SP task (interaction: *b* = −.26, *SE* =.10, *p* <.05) or baseline cortisol values (interaction: *b* = −.21, *SE* =.09, *p* <.05).

**Table 1:** Hierarchical ordinary least squares regression of cortisol reactivity on early parenting and recent traumatic events

	Model 1		Model 2	
	*b* (*SE*)	*B*	*b* (*SE*)	*B*
Receiving medication	−.06 (.10)	−.03	−.05 (.10)	−.02
Bedtime	.03 (.03)	.05	.03 (.03)	.06
Time initial saliva sample	.00 (.02)	−.00	.00 (.02)	−.01
Harsh, nonresponsive early parenting	−.04 (.08)	−.03	−.01 (.08)	−.01
Recent traumatic events	.11 (.05)	.12*	.13 (.05)	.14**
Harsh, nonresponsive parenting × recent traumatic events	–	–	−.28 (.10)	−.15**
Constant	.24 (.03)		.26 (.03)	
*R*^2^Δ			2.0%; *F* (1, 373) = 7.84, *p* <.01	
*R*^2^	1.7%; *F* (5, 373) = 1.31, *p* =.26		3.7%; *F* (6, 372) = 2.42, *p* <.05	

* *p* <.05;

** *p* <.01.

Simple effects were probed using the SPSS PROCESS macro (Hayes, 2013). Tests revealed the predicted effect of recent trauma in the group that experienced lower levels of harsh, nonresponsive parenting at age 3 (θ =.13, *SE* =.05, *p* <.01); the more traumatic events youth had recently experienced, the higher their cortisol reactivity. In contrast, among youth who experienced higher levels of harsh, nonresponsive parenting, the effect of recent trauma on cortisol reactivity was nonsignificant (θ = −.15, *SE* =.10, *p* =.14). Moreover, among youth who experienced two or more traumatic events in the past year, cortisol reactivity levels were significantly lower in the group of youth who experienced higher versus lower levels of harsh, nonresponsive parenting at age 3 years (θ = −.57, *SE* =.20, *p* <.01).

### Does the association between cortisol reactivity and children's externalizing and internalizing symptoms vary as a function of parenting in early childhood and recent exposure to traumatic events?

To test whether the effect of cortisol reactivity on children's internalizing and externalizing problems varied as a function of their developmental history of stressful relationships and experiences, we estimated three-way moderator analyses using the SPSS PROCESS macro (Hayes, 2013). All variables were mean-centered prior to analysis.

#### Externalizing problems

**Figure 2:**
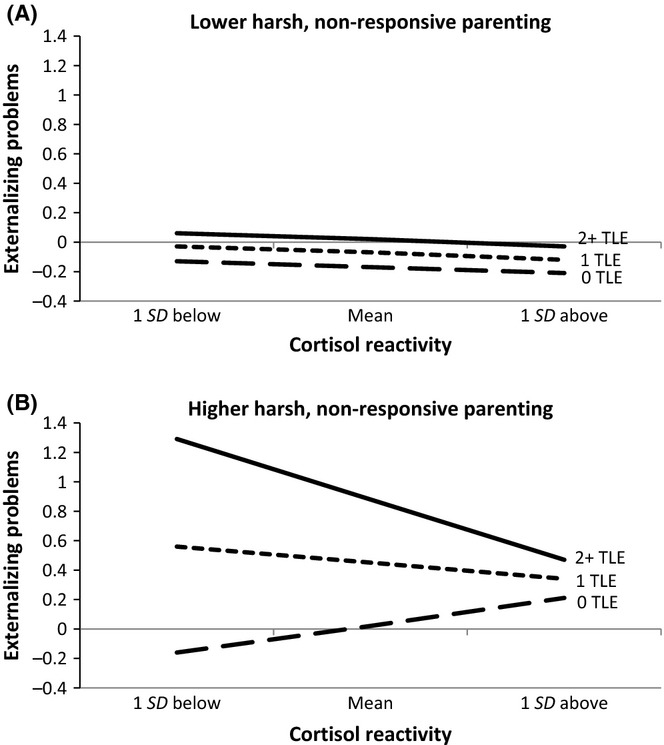
(A, B) Cortisol reactivity and recent traumatic events interact to predict externalizing problems when children have experienced higher (but not lower) levels of harsh, nonresponsive parenting

As shown in Table 2, there was a significant three-way interaction involving cortisol reactivity, harsh, nonresponsive parenting, and recent traumatic events, with the interaction accounting for an additional 1% of the variance in externalizing problems. Post hoc probing revealed that the two-way interaction between cortisol reactivity and traumatic events was significant for youth who had experienced higher levels of harsh, nonresponsive parenting (θ = −.45, *SE* =.19, *p* <.05), but not for youth who had experienced lower levels of harsh, nonresponsive parenting (θ =.00, *SE* =.10, *p* =.96; Figure 2A,B). Simple effects tests in the group that experienced higher levels of harsh, nonresponsive parenting revealed that cortisol reactivity was not associated with externalizing problems among those who had experienced zero (θ =.27, *SE* =.21, *p* =.19) or one (θ = −.16, *SE* =.14, *p* =.22) traumatic event in the past year. Among youth who had experienced two or more traumatic events, however, lower levels of cortisol reactivity were associated with higher levels of externalizing problems (θ = −.61, *SE* =.26, *p* <.05).

**Table 2:** Ordinary least squares regression analysis estimating three-way interaction

	Externalizing problems	Internalizing problems
	*b* (*SE*)	*b* (*SE*)
Harsh, nonresponsive parenting	.37 (.10)***	.16 (.10)
Recent traumatic events	.18 (.06)**	.11 (.06)
Cortisol reactivity	−.04 (.07)	−.02 (.07)
Male sex	.19 (.09)*	.10 (.08)
Child age	.01 (.00)	.01 (.00)
Household income	−.01 (.01)	−.01 (.01)
Parent education	−.07 (.04)*	−.06 (.04)
Parent occupational status	.00 (.02)	−.01 (.02)
Harsh, nonresponsive × traumatic event	.34 (.13)**	.19 (.13)
Harsh, nonresponsive × cort reactivity	.09 (.16)	.24 (.16)
Traumatic event × cort reactivity	−.12 (.09)	−.17 (.09)
Three-way interaction	−.44 (.22)*	−.71 (.22)**
Constant	−.58 (.02)	−.71 (.58)
*R*^2^	17.3%; *F* (12, 346) = 6.05, *p* <.001	11%; *F* (12, 346) = 3.57, *p* <.001

* *p* <.05;

** *p* <.01;

*** *p* <.001.

#### Internalizing problems

**Figure 3:**
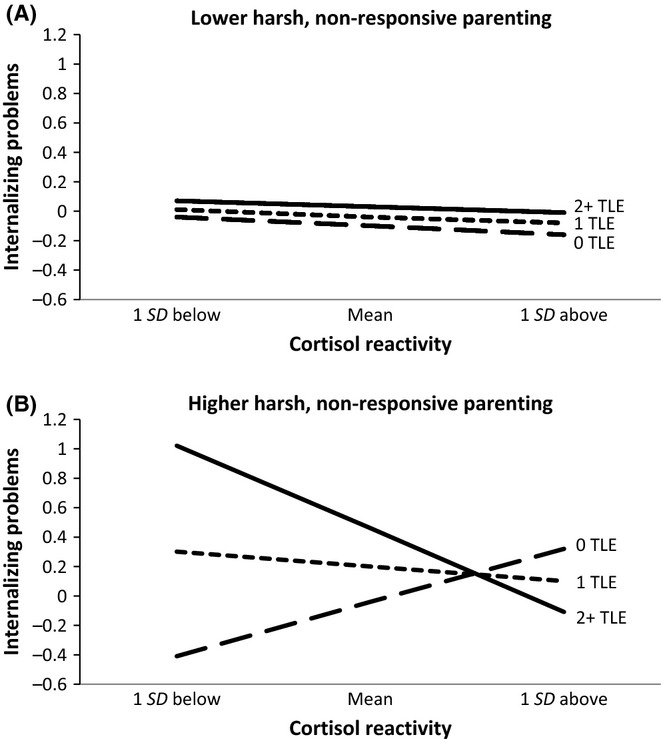
(A, B) Cortisol reactivity and recent traumatic events interact to predict internalizing problems when children have experienced higher (but not lower) levels of harsh, nonresponsive parenting

As shown in Table 2, there was a significant three-way interaction between cortisol reactivity, early parenting, and recent traumatic events, with the interaction accounting for 2.8% of the variance in internalizing problems. Post hoc probing revealed that the two-way interaction between cortisol reactivity and traumatic events was significant for youth who had experienced higher levels of harsh, nonresponsive parenting (θ = −.70, *SE* =.19, *p* <.001), but not for youth who had experienced lower levels of harsh, nonresponsive parenting (θ =.01, *SE* =.10, *p* =.90; Figure 3A,B). Simple effects tests in the group that experienced higher levels of harsh, nonresponsive parenting revealed that cortisol reactivity was not associated with internalizing problems among those who had experienced only one traumatic event in the past year (θ = −.15, *SE* =.14, *p* =.27). Among those who had not experienced any traumatic events, higher cortisol reactivity was associated with higher internalizing problems (θ =.55, *SE* =.21, *p* <.01), but among those who had experienced two or more traumatic events, lower levels of cortisol reactivity were associated with higher levels of internalizing problems (θ = −.85, *SE* =.26, *p* <. 01).

## Discussion

### Children's developmental history conditions cortisol reactivity to a psychosocial stressor

Consistent with the hypothesis that a developmental history of adversity would account for variation in cortisol reactivity, the effect of children's recent exposure to trauma depended on their early exposure to harsh, nonresponsive parenting. The more traumatic events children had recently experienced, the greater their cortisol reactivity to the social provocation task, but only if children had experienced lower levels of harsh, nonresponsive parenting in early childhood. The lowest levels of cortisol reactivity were observed among children who had experienced higher levels of harsh, nonresponsive parenting in early childhood and two or more recent traumatic events.

Because the traumatic events reported by children and caregivers most frequently reflected conflict and violence in the family and neighborhood, they may have captured harsh and nonresponsive parenting that was ongoing from early to middle childhood as well as other conflictual relationships within the family. To the extent that patterns of harsh and nonresponsive parenting were ongoing in CEDS families, the adverse effects of recent traumatic events may have been exacerbated by the absence of a warm, responsive caregiver who could adequately buffer youth from traumatic exposures (Hostinar, Sullivan, & Gunnar, 2014). The current findings provide a possible explanation for why exposure to stressful events is sometimes associated with hyper-reactivity of the HPA axis (Harkness et al., 2011; Linares et al., 2013) and other times associated with hypo-reactivity (Gunnar et al., 2009; MacMillan et al., 2009; Ouellet-Morin, Danese et al., 2011; Peckins, Dockray, Eckenrode, Heaton, & Susman, 2012; Saxbe et al., 2012; Shenk et al., 2010): the direction of these associations partly depends on the individual's history of exposure to harsh and nonresponsive relationships in early childhood and more recent exposure to traumatic events.

The pattern of heightened cortisol reactivity among children whose risk exposure was limited to the past year, but dampened reactivity among children who were exposed to harsh, nonresponsive parenting in early childhood as well as traumatic events in middle childhood, is consistent with theoretical accounts that highlight the biological costs related to long-term survival and reproduction that are involved when individuals maintain a state of behavioral and physiological vigilance (Del Giudice et al., 2011). The pattern is also consistent with data from a meta-analysis of basal cortisol levels and stressful experiences indicating that initial exposure to stressful experiences (e.g., combat exposure, physical violence) is associated with increased levels of cortisol output; however, over time, cortisol levels rebound to below-normal levels (Miller, Chen, & Zhou, 2007). One possibility is that relatively low levels of cortisol reactivity among individuals exposed to chronic stress reflect an adaptation at the level of the pituitary to repeated activation of the hypothalamus and release of corticotropin-releasing hormone (Heim, Ehlert, & Helhammer, 2000).

### Children's developmental history conditions the relationship between cortisol reactivity and internalizing and externalizing problems

Consistent with other evidence (Badanes et al., 2011; von Klitzing et al., 2012; Ouellet-Morin, Odgers et al., 2011), we found that when youth were exposed to harsh, nonresponsive parenting in early childhood as well as traumatic events in middle childhood, blunted cortisol reactivity was associated with elevated internalizing and externalizing problems. Consistent with a differential susceptibility framework (Belsky & Pluess, 2009, 2013), however, blunted cortisol reactivity was associated with very low levels of internalizing problems among children who had *not* experienced any recent traumatic events.

These behavior-problem findings highlight the importance of blunted cortisol reactivity as a risk factor for children's externalizing and internalizing problems, particularly when harsh and nonresponsive parent–child relationships in early childhood are followed by exposure to later traumatic events. The association between attenuated cortisol reactivity and internalizing symptoms may be specific to the prepubertal period. For example, in a sample of youth who had not yet made the transition to puberty, reductions in cortisol reactivity were associated with increases in internalizing symptoms, but the reverse was true once youth reached puberty (Hankin, Badanes, Abela, & Watamura, 2010). It is not clear why reductions in cortisol reactivity are associated with elevated internalizing (or externalizing) symptoms prior to adolescence, although it has been suggested that failure to mount an adequate stress response to psychosocial challenge could have adverse implications for children's ability to cope psychologically or behaviorally with the stressors in their lives, ultimately increasing risk for emotional and behavioral problems (Badanes et al., 2011).

### Limitations

Limitations of the study include the fact that cortisol values only increased 16%, on average, in response to the SP task. Although this effect was small, Kirschbaum and Helhammer (1989) have described ‘responders’ to psychosocial stress tests as those whose cortisol values increase by at least 15%. It is possible that efforts to elicit cortisol reactivity outside of a laboratory setting introduced noise (i.e., error) into the data that made it difficult to detect effects of the SP task. Other statistically significant effects were similarly small, including the interaction between parenting in early childhood and recent traumatic events, which accounted for only 2% of the variation in cortisol reactivity. Although relatively few studies report effect sizes for the association between parenting or traumatic events and cortisol reactivity, those that do describe similarly small effects (Marsman et al., 2012).

### Clinical implications

Pediatricians (in the United States) and general practitioners (in the United Kingdom) are at the front lines of those dealing with children and families. As such, there are growing calls for these clinicians to screen for children's exposure to stressful relationships and experiences, given what is known about the long-term consequences of childhood adversity on adult mental and physical health (Garner et al., 2012). Our findings suggest that alterations in HPA axis function may be one pathway through which stressful relationships and experiences increase children's risk for emotional and behavioral problems. Moreover, there is growing evidence that dysregulation of the HPA axis is implicated in physical health problems like cardiovascular disease and major depressive disorder via effects on the immune system (Miller, Maletic, & Raison, 2009; Ridker, 2007). Thus, clinicians who treat children should be able to recommend behavioral and psychological interventions that could minimize the risks to health associated with growing up in harsh, rejecting, and dangerous environments.

**Key points**

- Exposure to harsh, rejecting, or violent relationships in childhood is associated with alterations in HPA axis functioning; little is known about how the timing and chronicity of exposure to stressful experiences affect HPA axis function.
- Our data suggest that mixed findings in the literature on whether stressful experiences are associated with hyper- or hypo-reactivity of the HPA axis could be explained by individual differences in developmental history of exposure to stressful relationships and experiences.
- In the context of harsh and nonresponsive parent–child relationships in early childhood and exposure to traumatic events in middle childhood, low cortisol reactivity to psychosocial challenge was associated with elevations in children's internalizing and externalizing problems.

## Supporting information

**Figure S1** Children's Experiences and Development Study (CEDS) sampling frame. **Table S1** Differences between participants and eligible participants who refused to participate. **Table S2** Correlations among study variables. **Table S3** Means (standard deviations) for cortisol values (raw scores) in μg/dl. **Table S4** Differences between participants and nonparticipants from the recruitment sample.

## References

[b1] Badanes LS, Watamura SE, Hankin BL (2011). Hypocortisolism as a potential marker of allostatic load in children: Associations with family risk and internalizing disorders. Development and Psychopathology.

[b2] Belsky J, Melhuish E, Barnes J, Leyland AH, Romaniuk H, National Evaluation of Sure Start Research Team (2006). Effects of Sure Start local programmes on children and families: Early findings from a quasi-experimental cross sectional study. British Medical Journal.

[b3] Belsky J, Pluess M (2009). Beyond diathesis stress: Differential susceptibility to environmental influences. Psychological Bulletin.

[b4] Belsky J, Pluess M (2013). Beyond risk, resilience and dysregulation: Phenotypic plasticity and human development. Development and Psychopathology.

[b5] Bosch NM, Riese H, Reijneveld SA, Bakker MP, Verhulst FC, Ormel J, Oldehinkel AJ (2012). Timing matters: Long term effects of adversities from prenatal period up to adolescence on adolescents' cortisol stress response. The TRAILS study. Psychoneuroendocrinology.

[b6] Caldwell B, Bradley RH (1984). Home observation for measurement of the environment.

[b7] Caron A, Weiss B, Harris V, Catron T (2006). Parenting behavior dimensions and child psychopathology: Specificity, task dependency, and interactive relations. Journal of Clinical Child and Adolescent Psychology.

[b8] Danese A, McEwen BS (2012). Adverse childhood experiences, allostasis, allostatic load, and age-related disease. Physiology & Behavior.

[b9] Deater-Deckard K, Ivy L, Petrill SA (2006). Maternal warmth moderates the link between physical punishment and child externalizing problems: A parent – offspring behavior genetic analysis. Parenting, Science and Practice.

[b10] Del Giudice M, Ellis BJ, Shirtcliff EA (2011). The Adaptive Calibration Model of stress responsivity. Neuroscience and Biobehavioral Reviews.

[b11] Gadow KD, Sprafkin J (2005). Child and Adolescent Symptom Inventory-4 Revised (CASI-4R).

[b12] Garner AS, Shonkoff JP, Siegel BS, Dobbins MI, Earls MF, Garner AS, Wood DL (2012). Early childhood adversity, toxic stress, and the role of the pediatrician: Translating developmental science into lifelong health. Pediatrics.

[b13] German M, Gonzales NA, McClain DB, Dumka L, Millsap R (2013). Maternal warmth moderates the link between harsh discipline and later externalizing behaviors for Mexican American adolescents. Parenting, Science and Practice.

[b14] van Goozen SHM, Matthys W, Cohen-Kettenis PT, Gispen-de Wied C, Wiegant VM, van Engeland H (1998). Salivary cortisol and cardiovascular activity during stress in oppositional-defiant disorder boys and normal controls. Biological Psychiatry.

[b15] Gunnar MR, Frenn K, Wewerka SS, Van Ryzin MJ (2009). Moderate versus severe early life stress: Associations with stress reactivity and regulation in 10-12-year-old children. Psychoneuroendocrinology.

[b16] Gunnar M, Quevedo K (2007). The neurobiology of stress and development. Annual Review of Psychology.

[b17] Hankin BL, Badanes LS, Abela JRZ, Watamura SE (2010). Hypothalamic-pituitary-adrenal axis dysregulation in dysphoric children and adolescents: Cortisol reactivity to psychosocial stress from preschool through middle adolescence. Biological Psychiatry.

[b18] Harkness KL, Stewart JG, Wynne-Edwards KE (2011). Cortisol reactivity to social stress in adolescents: Role of depression severity and child maltreatment. Psychoneuroendocrinology.

[b19] Hayes AF (2013). Introduction to mediation, moderation, and conditional process analysis: A regression-based approach.

[b20] Heim C, Ehlert U, Helhammer DH (2000). The potential role of hypocortisolism in the pathophysiology of stress-related bodily disorders. Psychoneuroendocrinology.

[b21] Hostinar CE, Gunnar MR (2013). Future directions in the study of social relationships as regulators of the HPA axis across development. Journal of Clinical Child and Adolescent Psychology.

[b22] Hostinar CE, Sullivan RM, Gunnar MR (2014). Psychobiological mechanisms underlying the social buffering of the hypothalamic-pituitary-adrenocortical axis: A review of animal models and human studies across development. Psychological Bulletin.

[b23] Kirschbaum C, Helhammer DH (1989). Salivary cortisol in psychobiological research: An overview. Neuropsychobiology.

[b24] von Klitzing K, Perren S, Klein AM, Stadelmann S, White LO, Groeben M, Hatzinger M (2012). The interaction of social risk factors and HPA axis dysregulation in predicting emotional symptoms of five- and six-year-old children. Journal of Psychiatric Research.

[b25] Linares LO, Shrout PE, Nucci-Sack A, Diaz A (2013). Child maltreatment, dating perpetration of physical assault, and cortisol reactivity among disadvantaged female adolescents. Neuroendocrinology.

[b26] MacMillan HL, Georgiades K, Duku EK, Shea A, Steiner M, Niec A, Schmidt LA (2009). Cortisol response to stress in female youths exposed to childhood maltreatment: Results of the Youth Mood Project. Biological Psychiatry.

[b27] Marsman R, Nederhof E, Rosmalen JGM, Oldehinkel AJ, Ormel J, Buitelaar JK (2012). Family environment is associated with HPA-axis activity in adolescents. The TRAILS study. Biological Psychology.

[b28] McKee L, Roland E, Coffelt N, Olson AL, Forehand R, Massari C, Zens MS (2007). Harsh discipline and child problem behaviors: The roles of positive parenting and gender. Journal of Family Violence.

[b29] McLoyd VC, Smith J (2002). Physical discipline and behavior problems in African American, European American, and Hispanic children: Emotional support as a moderator. Journal of Marriage and the Family.

[b30] Miller GE, Chen E, Zhou ES (2007). If it goes up, must it come down? Chronic stress and the hypothalamic-pituitary-adrenocortical axis in humans. Psychological Bulletin.

[b31] Miller AH, Maletic V, Raison CL (2009). Inflammation and its discontents: The role of cytokines in the pathophysiology of major depression. Biological Psychiatry.

[b32] Ouellet-Morin I, Danese A, Bowes L, Shakoor S, Ambler A, Pariante CM, Arseneault L (2011). A discordant monozygotic twin design shows blunted cortisol reactivity among bullied children. Journal of the American Academy of Child and Adolescent Psychiatry.

[b33] Ouellet-Morin I, Odgers CL, Danese A, Bowes L, Shakoor S, Papadopoulos AS, Arseneault L (2011). Blunted cortisol responses to stress signal social and behavioral problems among maltreated/bullied 12-year-old children. Biological Psychiatry.

[b34] Peckins MK, Dockray S, Eckenrode JL, Heaton J, Susman EJ (2012). The longitudinal impact of exposure to violence on cortisol reactivity in adolescents. Journal of Adolescent Health.

[b35] Petersen AC, Crockett L, Richards M, Boxer A (1988). A self-report measure of pubertal status: Reliability, validity, and initial norms. Journal of Youth and Adolescence.

[b36] Ribbe D, Stamm BH (1996). Psychometric review of Traumatic Event Screening Instrument for Children (TESI-C). Measurement of stress, trauma, and adaptation.

[b37] Ridker PM (2007). Inflammatory biomarkers and risks of myocardial infarction, stroke, diabetes, and total mortality: Implications for longevity. Nutrition Reviews.

[b38] Saxbe DE, Margolin G, Shapiro LAS, Baucom BR (2012). Does dampened physiological reactivity protect youth in aggressive family environments?. Child Development.

[b39] Shenk CE, Noll JG, Putnam FW, Trickett PK (2010). A prospective examination of the role of childhood sexual abuse and physiological asymmetry in the development of psychopathology. Child Abuse & Neglect.

[b40] Smith JR, Brooks-Gunn J (1997). Correlates and consequences of harsh discipline for young children. Archives of Pediatrics & Adolescent Medicine.

[b41] Sroufe LA (2005). Attachment and development: A prospective, longitudinal study from birth to adulthood. Attachment & Human Development.

[b42] Straus MA, Hamby S, Finkelhor D, Moore D, Runyan D (1998). Identification of child maltreatment with the Parent-Child Conflict Tactics Scales: Development and psychometric data for a national sample of American parents. Child Abuse & Neglect.

[b43] Tarullo AR, Gunnar MR (2006). Child maltreatment and the developing HPA axis. Hormones and Behavior.

