# Supplementary material for: Interactive effects of early and recent exposure to stressful contexts on cortisol reactivity in middle childhood
Source: J Child Psychol Psychiatry. 2014 Jul 1;56(2):138–46. doi: 10.1111/jcpp.12287 (PMC4338758; doi:10.1111/jcpp.12287)

Supporting information for Jaffee et al., ***Interactive effects of early and recent exposure to stressful environments on cortisol reactivity in middle childhood***

Table S1. Differences between participants and eligible participants who refused to participate or could not be located on child, maternal, and family characteristics when children were 3 years old

| **Demographic Characteristics at Age 3** | Participated (n=400) | Refused or Not Located (n=729) | t (df) or Χ^2^ (df) |
| --- | --- | --- | --- |
| Household size | 4.20 (1.33) | 4.12 (1.57) | -.87 (1126), p = .39 |
| Mother’s age | 31.07 (6.30) | 30.09 (6.21) | -2.51 (1110), p < .05 |
| Child problem behaviors | 3.55 (6.07) | 3.35 (5.87) | -.54 (1078), p = .59 |
| Maternal malaise | 2.14 (2.01) | 2.02 (1.97) | -.99 (1016), p = .32 |
| No educational qualifications (mother) | 24% | 26% | Χ^2^ (1) = 1.00, p = .32 |
| No educational qualifications (father) | 27% | 33% | Χ^2^ (1) = 2.16, p = .15 |
| Unemployed (mother) | 64% | 70% | Χ^2^ (1) = 4.45, p < .05 |
| Unemployed (father) | 17% | 27% | Χ^2^ (1) = 8.45, p < .01 |
| Homeowner | 42% | 30% | Χ^2^ (1) = 16.28, p < .001 |
| Finding it hard to get by financially | 21% | 21% | Χ^2^ (1) = .00, p =.96 |
| Mother White ethnicity | 73% | 68% | Χ^2^ (1) = 2.66, p =.10 |
| Only English spoken at home | 74% | 67% | Χ^2^ (1) = 5.83, p < .05 |
| Harsh discipline | -.08 (.72) | -.07 (.74) | .28 (1127), p = .77 |
| Responsive parenting | 5.14 (1.44) | 5.02 (1.61) | -1.18 (1100), p = .24 |

Table S2. Correlations among Study Variables

|  | 1 | 2 | 3 | 4 | 5 | 6 | 7 | 8 | 9 | 10 | 11 | 12 | 13 |
| --- | --- | --- | --- | --- | --- | --- | --- | --- | --- | --- | --- | --- | --- |
| 1 Age | 1 |  |  |  |  |  |  |  |  |  |  |  |  |
| 2 Not White | .04 | 1 |  |  |  |  |  |  |  |  |  |  |  |
| 3 Male | -.02 | -.08 | 1 |  |  |  |  |  |  |  |  |  |  |
| 4 Income | .00 | -.20*** | -.01 | 1 |  |  |  |  |  |  |  |  |  |
| 5 Education | .04 | .04 | -.06 | .34*** | 1 |  |  |  |  |  |  |  |  |
| 6 Occ. Status | .00 | -.08 | -.04 | .56*** | .44*** | 1 |  |  |  |  |  |  |  |
| 7 Bedtime | .23*** | .26*** | -.01 | -.04 | .04 | .01 | 1 |  |  |  |  |  |  |
| 8 Saliva 1 time | -.07 | -.04 | .07 | -.07 | -.02 | -.10* | -.36*** | 1 |  |  |  |  |  |
| 9 HNR parenting | -.14** | .00 | .15** | -.16** | -.06 | -.14** | -.05 | .03 | 1 |  |  |  |  |
| 10 Traum Evnts | .05 | -.05 | .13** | -.09 | -.16** | -.09 | .00 | -.02 | .16 | 1 |  |  |  |
| 11 Cort React | -.07 | -.03 | -.01 | -.02 | -.02 | .00 | .05 | -.03 | -.02 | .12* | 1 |  |  |
| 12 EXT | .05 | -.09 | .15** | -.15** | -.17** | -.15** | -.03 | -.01 | .25*** | .23*** | -.05 | 1 |  |
| 13 INT | .07 | -.02 | .09 | -.12* | -.15** | -.13* | -.04 | -.03 | .13* | .17** | -.07 | .55*** | 1 |

Notes: Occ. Status = occupational status; HNR = harsh, non-responsive; Traum Evnts = recent traumatic events; Cort React = cortisol reactivity; EXT = externalizing symptoms; INT = internalizing symptoms

****p <* .001, ***p <* .01, **p <* .05

Table S3. Means (Standard Deviations) for Cortisol Values (Raw Scores) in µg/dl

|  | Minimum Value | 20 min post-task | 45 min post-task | 65 min post-task |
| --- | --- | --- | --- | --- |
| Completed Round 2 (n = 316) | .06 (.11) | .07 (.06) | .06 (.05) | .06 (.05) |
| Did Not Complete Round 2 (n = 66) | .09 (.23) | .12 (.27) | .08 (.11) | .07 (.07) |
| Total | .06 (.13) | .08 (.13) | .06 (.06) | .06 (.05) |

Table S4. Differences between participants and non-participants from the recruitment sample (regardless of eligibility for CEDS) on child, maternal, and family characteristics when children were 3 years old

| **Demographic Characteristics at Age 3** | Participated (n=400) | Non-Participants (n=6,270) | t (df) or Χ^2^ (df) |
| --- | --- | --- | --- |
| Household size | 4.20 (1.33) | 4.16 (1.48) | -.49 (6652), p = .63 |
| Mother’s age | 31.07 (6.30) | 29.78 (6.04) | -4.10 (6528), p < .001 |
| Child problem behaviors | 3.55 (6.07) | 3.78 (6.10) | .73 (6386), p = .47 |
| Maternal malaise | 2.14 (2.01) | 2.11 (2.05) | -.31 (6115), p = .76 |
| No educational qualifications (mother) | 24% | 27% | Χ^2^ (1) = 2.08, p = .15 |
| No educational qualifications (father) | 27% | 28% | Χ^2^ (1) = .02, p = .90 |
| Unemployed (mother) | 64% | 67% | Χ^2^ (1) = 1.05, p = .31 |
| Unemployed (father) | 17% | 23% | Χ^2^ (1) = 4.20, p < .05 |
| Homeowner | 42% | 36% | Χ^2^ (1) = 6.77, p < .01 |
| Finding it hard to get by financially | 21% | 18% | Χ^2^ (1) = 1.86, p = .17 |
| Mother White ethnicity | 73% | 78% | Χ^2^ (1) = 6.04, p < .05 |
| Only English spoken at home | 74% | 79% | Χ^2^ (1) = 6.19, p < .05 |
| Harsh discipline | -.08 (.72) | .00 (.75) | 2.25 (6079), p < .05 |
| Responsive parenting | 5.14 (1.44) | 4.97 (1.53) | -2.13 (6217), p < .05 |

Figure S1.

CEDS Sampling Frame


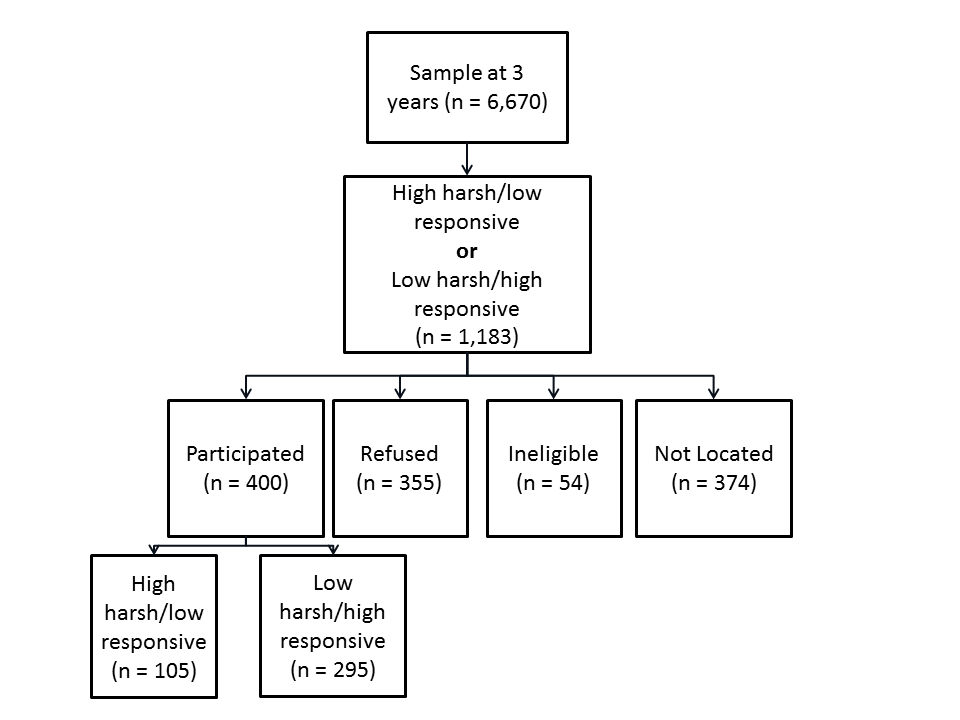

Supplement: Supplementary file 1 [file jcpp0056-0138-sd1.docx]
